# Trends in Global Burden of Alzheimer’s Disease and Other Dementias Attributable to High Fasting Plasma Glucose, 1990–2021

**DOI:** 10.3390/medicina60111783

**Published:** 2024-10-31

**Authors:** Irena Ilic, Vladimir Jakovljevic, Ivana Zivanovic Macuzic, Ana Ravic-Nikolic, Milena Ilic, Marija Sorak, Vesna Milicic

**Affiliations:** 1 Faculty of Medicine, University of Belgrade, 11000 Belgrade, Serbia; 2 Department of Physiology, Faculty of Medical Sciences, University of Kragujevac, 34000 Kragujevac, Serbia; 3 Department of Anatomy, Faculty of Medical Sciences, University of Kragujevac, 34000 Kragujevac, Serbia; 4 Department of Dermatovenerology, Faculty of Medical Sciences, University of Kragujevac, 34000 Kragujevac, Serbia; 5 Department of Epidemiology, Faculty of Medical Sciences, University of Kragujevac, 34000 Kragujevac, Serbia; 6 Department of Gynecology and Obstetrics, Faculty of Medical Sciences, University of Kragujevac, 34000 Kragujevac, Serbia

**Keywords:** Alzheimer’s disease, high fasting plasma glucose, burden, trends

## Abstract

*Background and Objectives*: Alzheimer’s disease and other dementias represent some of the leading public health concerns worldwide. This study aimed to assess the global burden of Alzheimer’s disease and other dementias attributable to high fasting plasma glucose in the last decades. *Materials and Methods*: A descriptive epidemiological study was conducted. The Global Burden of Disease (GBD) study data about deaths and Disability-Adjusted Life Years (DALYs) were used. All figures were presented as age-standardized rates (ASRs). The average annual percent change (AAPC) was computed using the Joinpoint regression analysis. Also, age-period-cohort analysis was performed. *Results*: A total of 2 million deaths from Alzheimer’s disease and other dementias were reported worldwide in 2021, whereby the total number deaths from Alzheimer’s disease and other dementias attributable to high fasting plasma glucose was 290,032 (98,900 males and 191,132 females) in 2021. The highest ASRs of burden of Alzheimer’s disease and other dementias attributable to high fasting plasma glucose were found in Afghanistan, Iraq, Morocco, Qatar, and the United States of America, while the lowest ASRs were in Belarus and Mongolia. From 1990 to 2021, a significant increase (*p* < 0.001) was noted in ASRs of deaths and DALYs for Alzheimer’s disease and other dementias attributable to high fasting plasma glucose. Looking at the GBD regions, the trends in ASRs for mortality and for DALYs of Alzheimer’s disease and other dementias attributable to high fasting plasma glucose between 1990 and 2021 showed a growth 10-fold faster in High-income North America (AAPC = 2.0%, for both equally) and Central Asia (AAPC = 2.4% and AAPC = 2.5%, respectively) than in the region of High-income Asia Pacific (AAPC = 0.1% and AAPC = 0.2%, respectively). The relative risk of mortality and DALYs for Alzheimer’s disease and other dementias attributable to high fasting plasma glucose demonstrated statistically significant (*p* < 0.0001) period and cohort effects, and net drift and local drifts. *Conclusions*: This study showed an increase in the global burden of Alzheimer’s disease and other dementias attributable to high fasting plasma glucose in the last decades. Future successful entire-population strategies targeting high fasting plasma glucose may reduce the burden of a wide range of these diseases.

## 1. Introduction

Alzheimer’s disease and other dementias are among the leading public health concerns, and considered as one of the most pressing health issues in the world. According to the World Health Organization (WHO) 2021 estimates, Alzheimer’s disease and other dementias ranked as the 7th leading cause of death worldwide in both males and females together, killing 1.8 million people [1]. Deaths due to Alzheimer’s disease and other dementias became the 4th leading cause of death in high-income countries in 2021 (behind ischemic heart disease, COVID-19, and stroke). In addition, Alzheimer’s disease and other forms of dementia feature highly in upper-middle-income countries (ranking as 6th among the leading causes of death) compared to other income groups, remaining the only group with this disease in the top 10 causes of death except high-income countries. Women accounted for 68% of deaths from Alzheimer’s and other forms of dementia. Dementia mainly occurs in older people, with increasing morbidity and mortality with age [1,2,3]. 

In addition to Alzheimer’s disease and other dementias continuing to be among the leading causes of mortality worldwide, dementias are a major cause of disability and dependency in the elderly, affecting memory, cognitive abilities, and behavior, ultimately interfering with one’s ability to perform daily activities. Consequently, dementias cause enormous negative effects on the lives and quality of life of individuals, families, caregivers, and medical and social care costs for the health care system and community [4,5,6]. It was estimated that globally in 2019, the cost of dementia was USD 1313.4 billion, corresponding to USD 23,796 per person with dementia [7]. Of the total global costs, informal care accounted for about 50%, while 16% were direct medical costs and 34% were direct social sector costs (including long-term care). However, although in 2019 61% of people with dementia lived in low-income and middle-income countries, 74% of the estimated dementia costs were in high-income countries [7].

According to the estimates of the Global Burden of Disease 2019 study, it is expected that globally, an increase in the number of persons who have dementia from 57.4 million in 2019 to 152.8 million in 2050 will be seen [3]. In 2020 and 2021, when COVID-19 emerged among the top ten causes of death, Alzheimer’s disease was the 7th among the leading causes of death in the United States and remained the 5th leading cause of death among Americans aged ≥65 [8]. Also, between 2000 and 2021, deaths from Alzheimer’s disease in the United States increased more than 140% [8]; the aging and population growth are considered as the largest contributors to this increase [9]. Although gender inequalities were observed among factors contributing to dementia in countries in Latin America and the Caribbean, it is unclear whether sex differences (in socioeconomic status, in education, in life expectancy, etc.) have direct/indirect effects on dementia frequency [10,11]. 

The most prevalent form of dementia is Alzheimer’s disease, contributing to an estimated 60–70% of cases [1]. Although age is the strongest established risk factor for dementia, the disease not only affects older people, but can also occur in people under the age of 65—the so-called “early-onset dementia”, which accounts for up to 10% of cases [1,12,13]. Some authors considered that the increase in the frequency of Alzheimer’s disease and other dementias attributable to high fasting plasma glucose is largely a reflection of population aging and growth worldwide, that can also be linked to a significant rise in diabetes mellitus prevalence in the world [14]. In the nationwide population-based cohort study in Korea, based on the National Health Insurance Service database (that collected data on 546,709 dementia-free individuals and followed them for 11 years), diabetes mellitus increased the risk of incidence and mortality both of early-onset and late-onset dementias [15]. However, epidemiological studies have reported that geographic differences in the burden of Alzheimer’s disease and other dementias attributable to high fasting plasma glucose could be due to differences in the prevalence of some underlying risk factors, i.e., high body mass index, smoking, alcohol use, dietary factors [9]. But to-date, the etiology of Alzheimer’s disease and other dementias remains insufficiently elucidated. 

In 2017, the WHO published a report that contains the “Global action plan on the public health response to dementia 2017–2025”, an important step forward in achieving well-being (physical, mental, and social) of persons who have dementia and their families and caregivers, who need to live a life with meaning and dignity [16]. Monitoring mortality trends of Alzheimer’s disease and other dementias attributable to high fasting plasma glucose can provide valuable insights into disease control and management. With the occurrence of the COVID-19 pandemic, numerous and enormous challenges also occurred towards achieving the Sustainable Development goals that the WHO and the United Nations set out in 2015 (i.e., reducing premature mortality from non-communicable diseases, including Alzheimer’s disease and other dementias attributable to high fasting plasma glucose, by one third by 2030, via the prevention, treatment, and promotion of the importance of mental health and well-being) [17]. A better understanding of the burden patterns of Alzheimer’s disease and other dementias attributable to high fasting plasma glucose worldwide can provide a further insight into potential means of more effectively preventing and managing this disease. This study aimed to assess the global burden of Alzheimer’s disease and other dementias attributable to high fasting plasma glucose in the last decades.

## 2. Materials and Methods

### 2.1. Study Design

In this research, with an epidemiological descriptive study design, data about Alzheimer’s disease and other dementias attributable to high fasting plasma glucose as underlying death cause were used to describe the global burden of disease in 1990–2021.

### 2.2. Data Source

The Global Burden of Disease (GBD) 2021 study data on Alzheimer’s disease and other dementias attributable to high fasting plasma glucose deaths were used [18]. The GBD 2021 is the largest, most comprehensive and up-to-date database involving epidemiological data for 204 countries and territories, and for 288 causes of death. The GBD 2021 study comprised a wide range of data sources (including vital registration, disease registries, censuses, health surveys, research studies, government reports, etc.). The GBD study represents a scientific effort conducted to estimate the magnitude and direction in trends of the burden of diseases and risk factors across countries. The GBD 2021 study provides a comprehensive comparative risk assessment framework, in order to determine the fraction of the disease burden attributable to 88 risk factors. Incorporating best practices of statistical modeling, the GBD study provides reliable estimates of health metrics, in accordance with the recommendations set out in the Guidelines for Accurate and Transparent Reporting of Health Assessments [19].

Based on the World Health Organization guidelines, the underlying cause of death includes a disease or injury that has started a series of diseases or an injury that has triggered a series of disease states that directly led to death. In this study, the Alzheimer’s disease and other dementias corresponded to the International Statistical Classification of Diseases and Related Health Problems, Ninth Revision (ICD-9) codes 290, 291.2, 291.8, 294, and 331, or by Tenth Revision (ICD-10) codes F00, F01, F02, F03, G30, and G31 [18,20]. According to the GBD 2021 study, as a risk factor specifically for Alzheimer’s disease and other dementias, high fasting plasma glucose was indicated [21].

### 2.3. Study Variables and Measures

This manuscript presents the burden of Alzheimer’s disease and other dementias attributable to high fasting plasma glucose, including mortality and Disability-Adjusted Life Years (DALYs). DALYs are computed as the sum of two components—years of life lost due to premature death (from that cause) and years of healthy life lost due to disability (from that cause). In this study, specific (age- and sex-specific) and age-standardized rates (expressed per 100,000 persons) were estimated. The direct standardization method, with the GBD standard population as the reference population, was used to compute the age-standardized rates (ASRs) [18].

In the current study, the burden of Alzheimer’s disease and other dementias attributable to high fasting plasma glucose is presented on global, regional (by 21 GBD regions and by 5 regions according to the Socio-demographic Index), and national levels (for 204 countries and territories). The results that were shown but not considered in comparisons (due to rates instability) are those for countries/territories with <100,000 population (i.e., Andorra, American Samoa, Antigua and Barbuda, Cook Islands, Dominica, Greenland, Marshall Islands, Monaco, Nauru, Niue, Northern Mariana Islands, Palau, Saint Kitts and Nevis, San Marino, Tokelau, Tuvalu); the results are presented, but not considered in the comparisons due to the instability of the rates. The Socio-demographic Index (SDI) indicates a country’s level of development, and is a summary measure of income per capita, average years of schooling in persons aged ≥15 years, and total fertility rate for females aged <25 [18]. Countries are categorized according to SDI quintiles as countries with low, low-medium, medium, high-medium, and high development levels. The GBD hierarchy of risk factors and accompanying exposure define the exposure of high fasting plasma glucose (>5.30 mmol/L) using a theoretical minimum risk exposure level at a fasting plasma glucose value of 4.88–5.30 mmol/L [21].

### 2.4. Statistical Analysis

Temporal trends in the burden of Alzheimer’s disease and other dementias attributable to high fasting plasma glucose were examined with the Joinpoint regression analysis (Joinpoint regression software, Version 4.9.0.0—March 2021, the Surveillance Research Program of the US National Cancer Institute), outlined by Kim et al. [22]. The magnitude and direction of trends were assessed by the Joinpoint regression analysis detecting temporal point(s) (so-called “joinpoint”), at which there was a significant change (increase or decrease) in the burden of Alzheimer’s disease and other dementias attributable to high fasting plasma glucose. The number of data points in this study was 32. The Monte Carlo Permutation test was used for multiple comparisons [22]. The Grid Search Method was used [23]. The minimal number of observations from a joinpoint to either end of the data (excluding the first or last joinpoint if it fell on an observation) was 2, and the minimal number of observations between two joinpoints (excluding any joinpoint if it fell on an observation) was 2, too. The settings for the number of points to place between adjacent observed k values in the Grid Search to something larger than the default of zero showed the same results. The program was run for 4499 permutations. Only the results based on the minimum number of joinpoints (i.e., trend as one straight line) were presented. Consequently, the Average Annual Percent Change (AAPC) was computed, with the corresponding 95% confidence interval (95% CI) [24]. Disparities in trends in the burden of Alzheimer’s disease and other dementias attributable to high fasting plasma glucose by sexes were tested using the comparability test [25]. The comparability test determines whether two line regression functions are parallel (test of parallelism). Trends were determined as significant (*p* < 0.05) according to the significance of the AAPC compared to zero. 

In addition, age-period-cohort analysis was performed to investigate the age, period, and birth cohort effects on the noted temporal trends. This analysis was done with the US NCI web-based statistical tool, using the method that Rosenberg et al. [26] proposed. The age-period-cohort analysis examined the effects of age, time period, and birth cohort on Alzheimer’s disease and other dementias attributable to high fasting plasma glucose using the numbers of deaths and corresponding person-years at risk for the selected age and sex groups and calendar time periods. Because no case of death of Alzheimer’s disease and other dementias attributable to high fasting plasma glucose happened in at least one year in the observed period, we omitted <40 age groups from the age-period-cohort analysis. In this study, those aged 40 and over years old were grouped into 5-year age groups (40–44, …, 90+), and 5-year time periods, i.e., 1991–1995, 1996–2000, 2001–2005, 2006–2010, 2011–2015, and 2016–2020 (with the period 1991–1995 as the reference period). In this study, 17 consecutive birth cohorts (from cohort 1896–1900 to cohort 1976–1980) were analyzed, whereby the birth cohort 1951–1955 was the reference cohort. In this study, the following parameters of the age-period-cohort analysis were presented: longitudinal age curves (representing the age-specific rates in the reference cohort, adjusted for period effect), period rate ratios (representing variations in mortality rates over time associated simultaneously with all age groups), cohort rate ratios (associated with changes in death rates across groups of individuals that have the same birth years, i.e., for successive age groups in successive time periods), and local drifts (representing the annual percentage changes for each age group over time) with net drift (representing the average annual percentage change in mortality per year of birth). The significance test that was used was a 1-df Wald test. Statistical significance was considered at *p* < 0.05.

### 2.5. Ethical Considerations

This study was conducted according to the guidelines of the Declaration of Helsinki, and it was approved by the Ethics Committee of the Faculty of Medical Sciences, University of Kragujevac (Ref. No.: No. 01-14321), on 30 November 2017. This study was conducted using publicly available data. Therefore, since our model-based analysis used aggregated and not individually identifiable data from publicly available sources, patients were not involved in the design, conduct, or reporting or dissemination plans of this research.

## 3. Results

**Figure 1:**
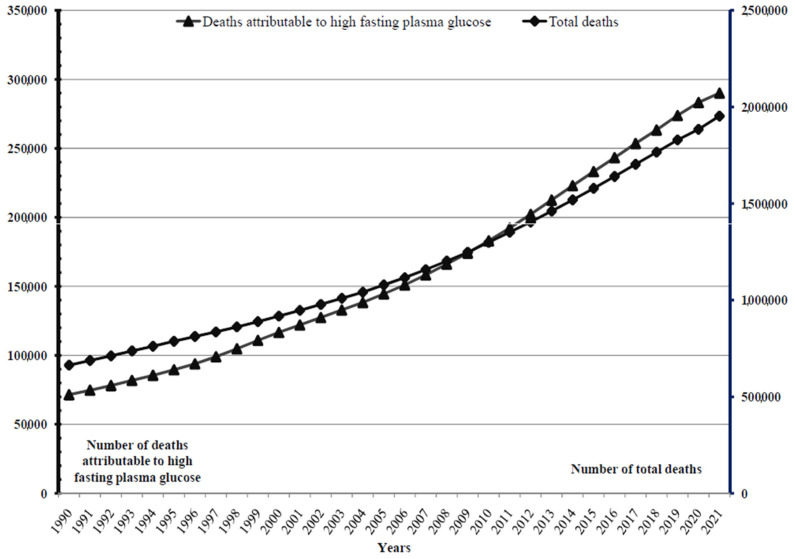
Alzheimer’s disease and other dementias: number of total deaths and deaths attributable to high fasting plasma glucose, in the world, 1990–2021.

A total of 37.2 million deaths from Alzheimer’s disease and other dementias were reported worldwide in the studied period 1990–2021; per year, the number of deaths ranged from 687,751 in 1990 to 1,952,677 in 2021 (Figure 1). A total of 5.2 million deaths from Alzheimer’s disease and other dementias attributable to high fasting plasma glucose were reported worldwide in the studied period 1990–2021; per year, the number of deaths ranged from 71,471 in 1990 to 290,032 in 2021. 

**Figure 2:**
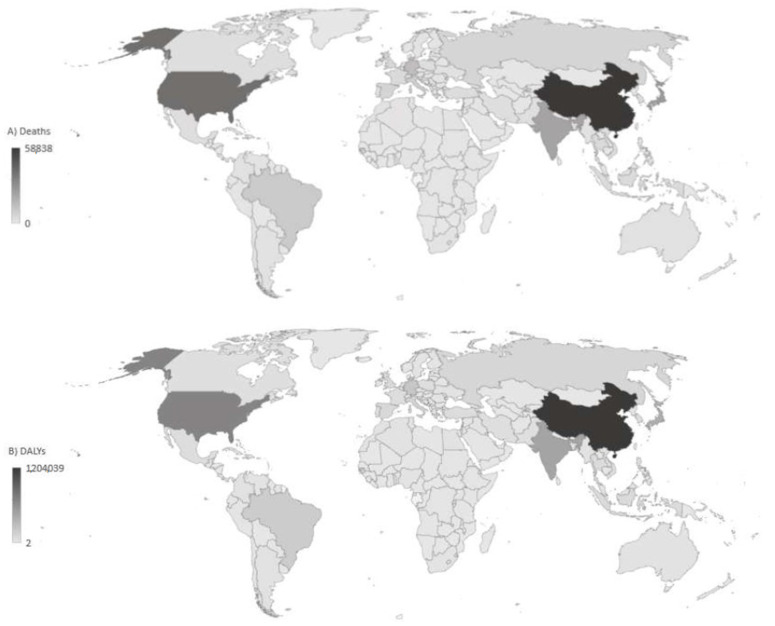
Number of deaths (**A**) and Disability-Adjusted Life Years—DALYs (**B**) of Alzheimer’s disease and other dementias attributable to high fasting plasma glucose, in all ages and both sexes together, by locations, 2021.

The most deaths (58,838) were observed in China, followed by the United States of America (40,511), Japan (26,028), India (22,525), and Germany (13,851) (Figure 2). The least number of Alzheimer’s disease and other dementias attributable to high fasting plasma glucose deaths (<10) was noted in some countries in Africa.

**Figure 3:**
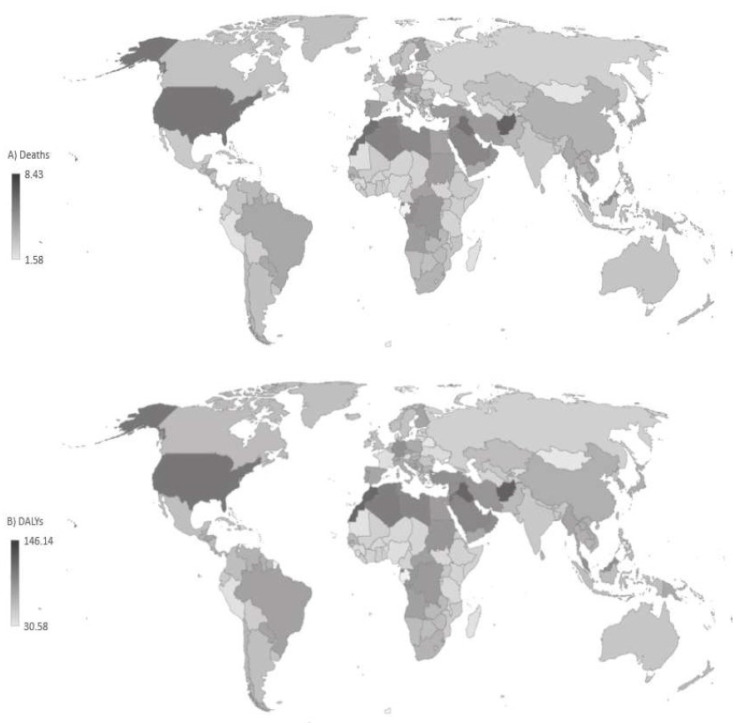
Age-standardized rates (ASRs, per 100,000) of deaths (**A**) and Disability-Adjusted Life Years—DALYs (**B**) of Alzheimer’s disease and other dementias attributable to high fasting plasma glucose, in all ages and both sexes together, by locations, 2021.

Globally, more deaths from Alzheimer’s disease and other dementias attributable to high fasting plasma glucose were noted in females (191,132) than males (98,900) in 2021 (female-to-male ratio was 1.93) (Table 1). The global ASR of mortality from Alzheimer’s disease and other dementias attributable to high fasting plasma glucose in both sexes together in 2021 was 3.7 per 100,000. The highest mortality ASRs were found in Afghanistan (6.8 per 100,000), followed by Qatar, Morocco, Iraq (equally about 6.2 per 100,000), and United States of America (6.0 per 100,000) (Figure 3). Belarus and Mongolia recorded the lowest rates (equally about 1.6 per 100,000).

The global ASR of DALYs of Alzheimer’s disease and other dementias attributable to high fasting plasma glucose in all ages and both sexes together in 2021 was 66.4 per 100,000 (Table 2). Also, the highest ASRs of DALYs of Alzheimer’s disease and other dementias attributable to high fasting plasma glucose were found in Afghanistan, Iraq, Morocco, and Qatar (equally about 115.0 per 100,000), while the lowest rates were reported in Belarus and Mongolia (about 30.0 per 100,000) (Figure 3).

Higher ASRs of mortality were recorded in women than in men, in the High SDI region than in other SDI regions, and in the region of High-income North America than in other GBD regions (Table 1). In both sexes, most of the Alzheimer’s disease and other dementias attributable to high fasting plasma glucose deaths (116,115; 40% of the total) were recorded in high SDI countries in 2021, whereby the highest ASRs were reported in the GBD region of High-income North America—5.66 per 100,000. The lowest ASR was evident in the region of Andean Latin America (2.07 per 100,000). 

From 1990 to 2021, the ASRs of mortality and DALYs for Alzheimer’s disease and other dementias attributable to high fasting plasma glucose showed a significant increase (*p* < 0.001) globally for both sexes in all ages, and across all SDI quintiles and all GBD regions (Table 1 and Table 2). According to the comparability test, the trends in burden due to Alzheimer’s disease and other dementias attributable to high fasting plasma glucose in males and females were parallel (the final selected model failed to reject parallelism, *p* > 0.05). The trends in ASRs both for deaths and DALYs for Alzheimer’s disease and other dementias attributable to high fasting plasma glucose from 1990 to 2021 showed a faster growth in Low–middle SDI countries compared to countries of other SDI quintiles. The trends in ASRs both for deaths and DALYs of Alzheimer’s disease and other dementias attributable to high fasting plasma glucose from 1990 to 2021 showed a 10-fold faster growth in the GBD region of High-income North America (AAPC = 2.0%, for both equally, and Central Asia (AAPC = 2.4% and AAPC = 2.5%, respectively) compared to the region of High-income Asia Pacific (AAPC = 0.1% and AAPC = 0.2%, respectively).

After adjusting for period and cohort effects, the risk of death and DALYs from Alzheimer’s disease and other dementias attributable to high fasting plasma glucose increased continuously with age in both sexes together (Table 3). The relative risk of death was 100 times higher in the age group of 90–94, compared to age group of 65–69 years. The net drift was 1.3% (95% CI = 1.1 to 1.4) per year for mortality, and the curves of local drift values were above 0 in all age groups, with one insignificant exception, the youngest age group (40–45 years). The relative risk of DALYs was 30 times higher in the age group of 90–94, compared to the age group of 65–69 years. The net drift was 1.2% (95% CI = 1.2 to 1.3) per year for DALYs, and the curves of local drift values were around 1 in all age groups. Period rate ratios increased over the entire period of this study (compared with 1991–1995, the rate of mortality and DALYs increased by 40% in 2016–2020). Cohort rate ratios for mortality and DALYs of Alzheimer’s disease and other dementias attributable to high fasting plasma glucose showed significantly upward patterns in all birth cohorts (compared with 1901–1905, the rate increased by 200% in 1976–1980): cohort effects constantly increased for mortality (RR from 0.5 [95% CI = 0.5 to 0.5] to 1.5 [95% CI = 0.7 to 3.0] during the entire study cohort), as well as for DALYs (RR from 0.5 [95% CI = 0.5 to 0.5] to 1.4 [95% CI = 1.2 to 1.7] during the entire study cohort). Results of Wald tests pointed out that the relative risk of mortality and DALYs for Alzheimer’s disease and other dementias attributable to high fasting plasma glucose showed statistically significant (*p* < 0.0001) cohort and period effects, and net drift and local drifts.

**Figure 4:**
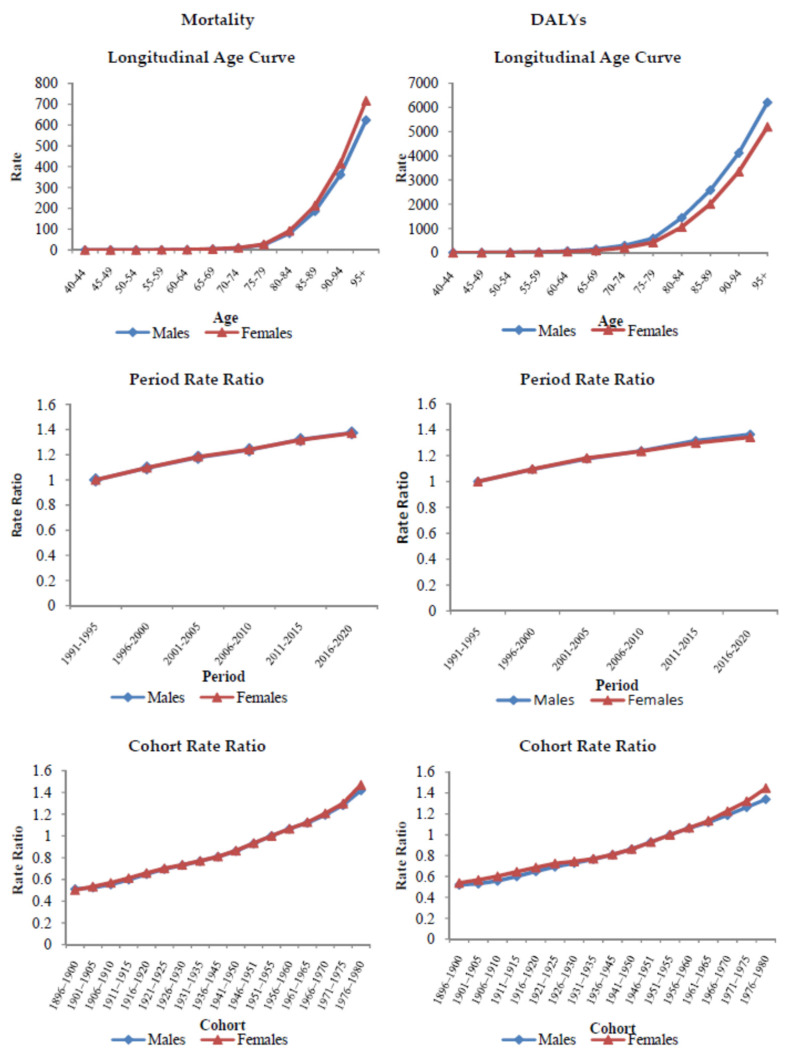
Global burden (mortality, Disability-Adjusted Life Years = DALYs) of Alzheimer’s disease and other dementias attributable to high fasting plasma glucose, by sexes, 1991–2020; an age-period-cohort analysis.

Globally, the risk of death and DALYs from Alzheimer’s disease and other dementias attributable to high fasting plasma glucose increased by age in both males and females, in particular after the age of 75 (Figure 4). The period effects demonstrated an upward pattern since 1991 both in men and in women. The risk of deaths and DALYs from Alzheimer’s disease and other dementias attributable to high fasting plasma glucose increased, in general, with birth cohort in both sexes. The local drift values were around 1 in all age groups in both sexes, while a non-significant value was noted in the 40–45 age group both in men and women. For mortality, the net drift was 1.3% (95% CI = 1.1 to 1.5) in men, while in women it was 1.3% (95% CI = 1.1 to 1.4). For DALYs, the net drift was 1.2% (95% CI = 1.2 to 1.3) both in men and in women. The Wald test indicated statistically significant period and cohort effects for both sexes, and net drift and local drifts (*p* < 0.0001).

## 4. Discussion

There were apparent discrepancies in the burden of Alzheimer’s disease and other dementias attributable to high fasting plasma glucose in 2021: higher age-standardized rates were observed in women than in men, in the high-SDI region than in other SDI quintiles, and in the GBD High-income North America region than in other GBD regions. Growth trends in the burden of Alzheimer’s disease and other dementias attributable to high fasting plasma glucose in the observed period were the slowest in the High-income Asia Pacific region. 

The differences across countries by ASRs of mortality and DALYs for Alzheimer’s disease and other dementias attributable to high fasting plasma glucose in both sexes together were approximately threefold in 2021 (for example: in the High-income GBD regions versus Andean Latin America, Eastern Europe, and Sub-Saharan Africa; or Qatar and the United States of America versus Belarus and Mongolia). Geographic variations in the mortality of Alzheimer’s disease and other dementias attributable to high fasting plasma glucose might exist due to the different age structures of different countries’ populations, increased life expectancy, availability of health care services, the improvements in therapeutic modalities, and differences in the implementation of prevention strategies, as well as in practices of dementia certification and registration [27,28,29]. In addition, international variations in dementia could partly be related to geographic differences in the prevalence of genetic factors, as suggested by numerous studies and meta-analyses about dementia and its relation to genetic variations in global regions such as America, Europe, Asia, and Africa [30,31,32,33,34,35]. In addition, the differences in burden observed across the SDI quintiles might be due to various social, economic, and healthcare-related factors that include literacy, education, occupational attainment, and other factors that contribute to cognitive reserve, which itself might differ across diverse cultures [36]. The life expectancy is increasing, which is one of the particular drivers of aging in high-income countries, and research shows that the risk of mortality is 1.56–5.69 times higher among older persons living with dementia [37]. Also, a higher awareness of dementia in high-income countries is present and an underestimation of the dementia burden, particularly in low- and middle-income countries, cannot be excluded [36,37].

In the last decades, a significant increase in the burden of Alzheimer’s disease and other dementias attributable to high fasting plasma glucose was recorded globally in both sexes and all ages, as well as in all SDI regions and all GBD regions. Many factors, like demographic aging and growth, increased life expectancy, improved healthcare coverage, and updated standards for diagnostics, take part in the noted increasing trends [38,39]. In the present study, the increasing effect of age on the global burden (mortality and DALYs) of Alzheimer’s disease and other dementias attributable to high fasting plasma glucose was observed continuously. The increasing effect of age might be due to older patients possibly experiencing less opportunities for effective care than those of younger age, as well as by complications of high fasting plasma glucose/diabetes (including cardiovascular disease, blindness, etc.) being more frequent in old individuals compared to younger persons [40,41]. According to the GBD 2021 study, the share of older persons aged 70 and above was 6.3% of the 7900 million total world population in 2021, while this proportion was 3.8% of the 5300 million total world population in 1990 [18]. On the other hand, the prevalence of diabetes mellitus (defined as ‘Self-report and/or medical treatment for diabetes, or fasting blood glucose ≥ 126 mg/dL (≥7.0 mmol/L)’) is increasing worldwide in the last decades, as is the evidence of the elevated high frequency of Alzheimer’s disease and other dementias [42,43]. Both Alzheimer’s disease and vascular dementia were more common in individuals with diabetes than in those without diabetes [44], whereby diabetes increased incident dementia risk an additional 35% in APOEε4 carriers (RR = 1.35, 95% CI = 1.13–1.63) [45]. Although mechanisms that would explain the link between high fasting plasma glucose and Alzheimer’s disease and other dementias were not fully understood, some authors suggested that vascular disease and alterations in glucose, insulin, mitochondrial dysfunction, hippocampal and amygdalar atrophy, inflammation, oxidative stress, and amyloid-β protein and tau protein metabolism underlie the pathophysiology; thus, recently it was put forward that Alzheimer’s disease can be regarded as “type 3 diabetes” [14,46,47,48,49]. In contrast, one study from Sweden showed that in persons ≥ 75 years, diabetes did not show a significant relationship with Alzheimer’s disease, but was associated with vascular dementia [50]. Still, despite some pathological mechanisms (cellular and molecular) being shared between Alzheimer’s disease and diabetes, the mediating effect of blood glucose levels on the occurrence of Alzheimer’s disease is still not completely known [51]. This is of particular interest given that a recent large study in the United States showed that even in patients without diabetes, higher blood glucose levels significantly increased the risk of dementia (adjusted hazard ratio for dementia 1.18 [95% CI 1.04–1.33]), with the risk increasing with increasing blood glucose levels [52]. According to the results of a meta-analysis of case–control studies, levels of fasting blood glucose were higher in patients with Alzheimer’s disease than in controls [51]. These results point out that patients with Alzheimer’s disease have an impaired glucose metabolism. In the circumstances of increased blood glucose levels and decreased sensitivity of insulin receptors, the risk of Alzheimer’s disease might increase due to the disfunction of mitochondria in nerve cells and the inflammatory response, while high blood glucose levels can also promote the phosphorylation of the tau protein and lead to neuronal damage [51,53,54]. Additionally, several studies have shown that elevated fasting plasma glucose levels could be associated with the pathophysiology of Alzheimer’s disease in nondiabetic persons [49,55], independent of vascular risk factors for dementia [56], and could be a risk factor for mild cognitive impairment that carries a high risk of transition to dementia [57]. Nevertheless, future studies need to investigate this complex interplay between high blood glucose levels and the risk of dementia.

Also, in the current study, the period effect on the global burden (mortality and DALYs) of Alzheimer’s disease and other dementias attributable to high fasting plasma glucose shows an increase over time. The observed trend could be due to the development of the global economy, which was accompanied with a change in diets, such as the consumption of food high in sugar and fat, as well as the decline in physical activity brought about by urbanization, air pollution, and obesity [58]. There is also considerable heterogeneity in the observed period in the progress of the management both of high fasting plasma glucose and Alzheimer’s disease and other dementias across countries, the implementation of preventive measures, the availability and effectiveness of therapy [59,60]. 

In addition, the rate ratio for mortality and DALYs of Alzheimer’s disease and other dementias attributable to high fasting plasma glucose showed a continuous increasing trend with increasing birth cohort. The cohort effects increased continuously from the 1896–1900 birth cohorts to more recent birth cohorts, which indicated an increased risk of mortality in the younger generations, both in males and females. The increased cohort effects observed in young generations (after the 1951–1955 birth cohorts) could be due to the fact that younger generations (born after World War II) with improving socioeconomic circumstances showed changing lifestyles that promote unhealthy eating habits, sedentary behavior, and obesity [61,62].

In this study, the global burden of Alzheimer’s disease and other dementias attributable to high fasting plasma glucose in 2021 was higher in women than in men across all ages, in particular in the oldest. Nearly two-thirds of deaths from Alzheimer’s disease and other dementias attributable to high fasting plasma glucose in 2021 occurred in women. A recent systematic review with a meta-analysis revealed that the only sex difference in the prevalence of any dementia occurred in the 85+ age group, and that no sex difference was recorded for vascular dementia, while Alzheimer’s disease was more prevalent in women among adults aged 65+/90+ [11]. Differences in the global burden of dementia by sex can be linked either with the influence of biological differences (differences in hormones and metabolism) on the risk of dementia, or with the contribution of numerous socio-demographic factors such as differences in education level, occupation, vascular risk factors, and social support [63]. For example, post-menopausal women experience increased insulin resistance because the levels of estrogen decrease, thus in turn increasing their risk of Alzheimer’s disease and other dementias [64]. In addition, life expectancy at birth in 2021 for males was estimated at 69 years, while life expectancy at birth in 2021 for females was estimated at 74 years [65]. The average survival after dementia diagnosis varied between 4 and 8 years, whereby male sex was detrimental to Alzheimer’s disease patient survival [66].

Additionally, systematic reviews suggested that people with Alzheimer’s disease and other dementias are at high risk of getting COVID-19 and having a poor outcome once infected [67,68,69]. Some studies indicated that cases with Alzheimer’s disease have an increased risk of infection with SARS-CoV-2 due both to the level of angiotensin-converting enzyme 2 in the brain tissue and the promotion of amyloid beta generation due to the infection itself [70]. During the early phase of the COVID-19 pandemic (from March through December 2020) in the United States of America, among persons with Alzheimer’s disease and related dementias, it was reported that the excess mortality was 25.7%, compared with the same period in 2019 [71]. Analysis of post-mortem brain tissue in cases of Alzheimer’s disease who survived a COVID-19 infection (COVID-AD) revealed a significant loss of microglial homeostasis, reduced cortical astrocyte numbers, and dysregulation of oligodendrocyte and myelination pathways in the hippocampus in COVID-AD patients as opposed to non-COVID-AD patients [72]. Although the relationship between Alzheimer’s disease and the COVID-19 pandemic received great attention, the question remains what the total consequences of the COVID-19 pandemic will be on Alzheimer’s disease and other dementias attributable to high fasting plasma glucose burden, whereby the additional effect of long COVID-19 on the burden of these diseases will be evaluated in future studies [68,70].

Apart from that, it is still unclear to what extent variations among countries, with regard to strategies of prevention, differences in genetic factors, and being exposed to certain still unknown factors, contribute to the global burden of Alzheimer’s disease and other dementias attributable to high fasting plasma glucose [28,29,30,34]. Thus, the diagnostics and registration of Alzheimer’s disease and other dementias attributable to high fasting plasma glucose still need to be improved further, so that improved estimations of epidemiological patterns among subtypes of dementias can be possible. Evaluating the distribution and magnitude of growth in the burden of Alzheimer’s disease and other dementias is crucial to develop targeted public health policies and a rational allocation of resources.

### 4.1. Strengths and Limitations of the Research

The present study provides estimates at the global, regional, and country levels, covering a span of 32 years, in such a way as to measure the age, period, and cohort effects on the burden of Alzheimer’s disease and other dementias attributable to high fasting plasma glucose through time. However, some limitations of this study should be considered. First, data reliability, accuracy, and coverage regarding the mortality of Alzheimer’s disease and other dementias attributable to high fasting plasma glucose always represent a question that can be raised. Namely, potential sources of bias in estimating trends in the burden of Alzheimer’s disease and other dementias attributable to high fasting plasma glucose worldwide may include inconsistent availability and variable quality of data, use of information collected from some self-reported data (which may include measurement uncertainty), information obtained from samples that are not representative of the entire country, failure to capture associated comorbidities in some populations, or the issue of under-reporting the burden of Alzheimer’s disease and other dementias attributable to high fasting plasma glucose levels, particularly in low-income countries. Finally, an epidemiological fallacy that is inherent in the correlation study design represents a large issue with this research. Thus, the findings presented in this research regarding the burden of Alzheimer’s disease and other dementias attributable to high fasting plasma glucose must be elucidated in analytical longitudinal studies.

### 4.2. Implications of the Research

Assessing the magnitude and direction of trends in the burden of Alzheimer’s disease and other dementias attributable to high fasting plasma glucose is of great public health importance. However, further efforts are needed to improve diagnostics and disease registration to continuously monitor trends in the burden of Alzheimer’s disease and other dementias attributable to high fasting plasma glucose, particularly in resource-limited countries. Firstly, a considerable number of people could be experiencing a burden from Alzheimer’s disease and other dementias attributable to high fasting plasma glucose, especially in high-SDI countries, in elderly and females. Since until now there has been no effective therapy for dementia, the key strategy to reduce the burden of this disease is to identify new, and address the known, modifiable risk factors in order to prevent the development of the condition. Therefore, in addition to the necessity of further research in order to find successful therapeutic modalities [73], it is also necessary to plan an increase in the use of health and social care services for patients with dementia [3,74], as well as resources to support caregivers of people with dementia [75]. As some studies have reported a decrease in the incidence of dementia in the most developed countries in North America and Europe [76], suggesting that this may be a consequence of successfully implemented prevention programs for cardiovascular, metabolic, and other diseases, further possibilities in reducing the burden of dementia include improvements in the management of risk factors for dementia, increasing the level of education, and raising public awareness. Since regions with higher SDIs were more affected by the burden of Alzheimer’s disease and other dementias attributable to high fasting plasma glucose than regions with low SDIs, it is necessary to carry out risk factor screening (for high fasting plasma glucose) and early diagnosis and treatment for the associated conditions or diseases in the exposed people in these populations. Since the elderly and females represent particularly vulnerable subgroups of the population, especially when they have associated conditions, diseases, and other risk factors (such as obesity, arterial hypertension, low physical activity, deafness, etc.), policy makers and health professionals should provide resources to improve the health care system for the elderly and women.

## 5. Conclusions

The increasing trends in the burden of Alzheimer’s disease and other dementias attributable to high fasting plasma glucose were observed worldwide in last decades. The most unfavorable trends were recorded in high-SDI regions, especially in the region of High-income North America. With the aging of the population, the burden of Alzheimer’s disease and other dementias attributable to high fasting plasma glucose is becoming a global health problem. The burden of this disease affects the elderly, with the greatest increase in rates over time observed in the 70+ age group. In addition, Alzheimer’s disease and other dementias attributable to high fasting plasma glucose especially affect females, with disease burden rates remaining almost twice as high in females than in males. The findings of this study emphasize the urgent need for more effective prevention and management strategies. The relationship of Alzheimer’s disease and other dementias and high fasting plasma glucose needs to be further investigated in analytical research studies. Future successful entire-population strategies targeting high fasting plasma glucose may reduce the burden of a wide range of these diseases.

## Figures and Tables

**Table 1:** Mortality of Alzheimer’s disease and other dementias attributable to high fasting plasma glucose, in all ages, by locations, 1990–2021; a joinpoint regression analysis.

	Deaths in 1990	ASR in 1990	Deaths in 2021	ASR in 2021	AAPC (ASR) (95% CI)	*p* Value
Global						
Male	23,067	2.30	98,900	3.26	1.2 * (1.2 to 1.3)	<0.001
Female	48,404	2.83	191,132	4.03	1.2 * (1.1 to 1.3)	<0.001
Both sex	71,471	2.64	290,032	3.73	1.2 * (1.1 to 1.3)	<0.001
— SDI regions						
High SDI	30,545	2.88	116,115	4.27	1.4 * (1.2 to 1.5)	<0.001
High–middle SDI	17,556	2.61	65,959	3.54	1.1 * (0.9 to 1.2)	<0.001
Middle SDI	14,869	2.66	69,335	3.42	0.9 * (0.8 to 0.9)	<0.001
Low–middle SDI	6394	1.92	30,601	3.19	1.7 * (1.7 to 1.8)	<0.001
Low SDI	2025	1.91	7757	2.83	1.3 * (1.3 to 1.4)	<0.001
— GBD regions						
Andean Latin America	193	1.21	1124	2.07	1.8 * (1.7 to 2.0)	<0.001
Australasia	470	2.26	1954	3.00	1.0 * (0.9 to 1.1)	<0.001
Caribbean	477	2.34	1576	2.77	0.5 * (0.5 to 0.5)	<0.001
Central Asia	491	1.39	1477	2.67	2.4 * (2.3 to 2.6)	<0.001
Central Europe	2626	2.39	8628	3.52	1.3 * (1.2 to 1.3)	<0.001
Central Latin America	1413	2.40	6645	2.91	0.5 * (0.4 to 0.5)	<0.001
Central Sub-Saharan Africa	286	3.53	1125	4.70	0.9 * (0.8 to 1.0)	<0.001
East Asia	13,420	3.31	61,212	3.62	0.4 * (0.2 to 0.6)	<0.001
Eastern Europe	2969	1.46	8181	2.27	1.6 * (1.6 to 1.7)	<0.001
Eastern Sub-Saharan Africa	659	2.02	2282	2.57	0.8 * (0.8 to 0.9)	<0.001
High-income Asia Pacific	5807	3.78	29,848	4.00	0.1 * (0.1 to 0.2)	<0.001
High-income North America	11,460	3.09	43,128	5.66	2.0 * (2.0 to 2.4)	<0.001
North Africa and Middle East	3068	3.20	14,262	4.89	1.5 * (1.4 to 1.6)	<0.001
Oceania	41	3.60	140	4.01	0.3 * (0.3 to 0.3)	<0.001
South Asia	5029	1.71	27,989	2.87	1.7 * (1.7 to 1.8)	<0.001
Southeast Asia	3448	2.40	15,380	3.58	1.3 * (1.2 to 1.4)	<0.001
Southern Latin America	734	1.99	2949	3.15	1.5 * (1.5 to 1.5)	<0.001
Southern Sub-Saharan Africa	475	2.64	1270	3.56	1.1 * (1.0 to 1.3)	<0.001
Tropical Latin America	1955	3.31	9628	4.04	0.8 * (0.8 to 0.9)	<0.001
Western Europe	15,706	2.69	48,690	3.71	1.1 * (1.0 to 1.1)	<0.001
Western Sub-Saharan Africa	742	1.55	2545	2.36	1.5 * (1.4 to 1.5)	<0.001

ASR = Age-standardized rate (per 100,000); For full period (1990–2021) presented AAPC = Average Annual Percentage Change; 95% CI = Confidence Interval; SDI = Socio-demographic Index. * Statistically significant trend (*p* < 0.05). Source: Global Burden of Disease study [18].

**Table 2:** DALYs of Alzheimer’s disease and other dementias attributable to high fasting plasma glucose, in all ages, by locations, 1990–2021; a Joinpoint regression analysis.

	DALYs (Number) in 1990	ASR in 1990	DALYs (Number) in 2021	ASR in 2021	AAPC (ASR) (95% CI)	*p* Value
Global						
Male	497,842	41.30	1,929,129	57.71	1.2 * (1.1 to 1.3)	<0.001
Female	943,698	51.33	3,419, 725	72.55	1.2 * (1.1 to 1.3)	<0.001
Both sex	1,441,540	47.07	5,348,854	66.42	1.2 * (1.1 to 1.3)	<0.001
— SDI regions						
High SDI	565,750	51.18	1,910,976	75.11	1.3 * (1.2 to 1.4)	<0.001
High–middle SDI	357,816	46.12	1,237,594	64.33	1.2 * (1.0 to 1.3)	<0.001
Middle SDI	327,924	48.47	1,411,109	63.35	0.9 * (0.8 to 1.0)	<0.001
Low–middle SDI	142,662	35.92	622,127	57.36	1.5 * (1.5 to 1.6)	<0.001
Low SDI	45,683	34.41	162,087	49.47	1.2 * (1.2 to 1.2)	<0.001
— GBD regions						
Andean Latin America	3924	23.22	21,937	39.74	1.8 * (1.7 to 2.0)	<0.001
Australasia	9184	41.29	33,122	53.45	0.9 * (0.9 to 1.0)	<0.001
Caribbean	10,332	45.81	30,087	54.44	0.5 * (0.5 to 0.5)	<0.001
Central Asia	10,000	26.09	30,980	50.89	2.5 * (2.4 to 2.6)	<0.001
Central Europe	56,073	44.82	160,790	65.91	1.3 * (1.2 to 1.3)	<0.001
Central Latin America	32,488	49.62	137,130	59.09	0.4 * (0.4 to 0.5)	<0.001
Central Sub-Saharan Africa	6810	61.21	24,293	80.90	0.9 * (0.8 to 1.0)	<0.001
East Asia	294,748	56.22	1,246,621	66.37	0.6 * (0.4 to 0.8)	<0.001
Eastern Europe	63,685	27.35	156,198	42.84	1.6 * (1.6 to 1.7)	<0.001
Eastern Sub-Saharan Africa	13,916	34.28	45,666	43.80	0.8 * (0.8 to 0.9)	<0.001
High-income Asia Pacific	106,880	63.12	451,644	68.17	0.2 * (0.1 to 0.3)	<0.001
High-income North America	3924	57.20	731,687	99.65	2.0 * (1.8 to 2.2)	<0.001
North Africa and Middle East	213,369	59.36	300,182	91.57	1.5 * (1.5 to 1.6)	<0.001
Oceania	66,256	67.24	3296	75.67	0.4 * (0.3 to 0.4)	<0.001
South Asia	1032	32.12	569,302	51.08	1.5 * (1.5 to 1.5)	<0.001
Southeast Asia	115,263	44.42	309,894	64.21	1.2 * (1.1 to 1.3)	<0.001
Southern Latin America	74,194	37.22	53,986	58.30	1.4 * (1.4 to 1.5)	<0.001
Southern Sub-Saharan Africa	15,032	47.16	26,566	64.33	1.2 * (1.1 to 1.3)	<0.001
Tropical Latin America	9356	61.55	185,362	76.27	0.9 * (0.8 to 0.9)	<0.001
Western Europe	41,886	46.35	779,500	63.81	1.0 * (1.0 to 1.1)	<0.001
Western Sub-Saharan Africa	282,168	26.33	50,610	40.06	1.5 * (1.4 to 1.5)	<0.001

DALYs = Disability-Adjusted Life Years; ASR = Age-standardized rates (per 100,000); For full period (1990–2021) presented AAPC = Average Annual Percentage Change; 95% CI = Confidence Interval; SDI = Socio-demographic Index. * Statistically significant trend (*p* < 0.05). Source: Global Burden of Disease study [18].

**Table 3:** Age, period, and cohort effects on global burden (mortality, DALYs) of Alzheimer’s disease and other dementias attributable to high fasting plasma glucose, 1991–2020.

		Mortality		DALYs	
		Effect	95% CI	Effect	95% CI
Age *					
	40–44	0.0	0.0–0.0	0.2	0.2–0.2
	45–49	0.0	0.0–0.0	2.1	2.0–2.1
	50–54	0.1	0.1–0.1	8.7	8.6–8.9
	55–59	0.5	0.5–0.5	27.3	27.0–27.6
	60–64	1.7	1.6–1.7	72.4	71.8–73.1
	65–69	4.5	4.4–4.5	163.3	162.0–164.5
	70–74	10.7	10.5–10.9	333.1	330.0–336.2
	75–79	27.0	26.5–27.6	678.3	671.9–684.7
	80–84	91.1	89.3–92.9	1653.8	1638.1–1669.7
	85–89	213.6	209.4–217.8	2987.0	2957.6–3016.7
	90–94	414.8	406.6–423.3	4743.9	4694.0–4794.3
	95+	715.7	700.7–731.0	7135.7	7049.5–7223.0
Period					
	1991–1995	1.0	1.0–1.0	1.0	1.0–1.0
	1996–2000	1.1	1.1–1.1	1.1	1.1–1.1
	2001–2005	1.2	1.2–1.2	1.2	1.2–1.2
	2006–2010	1.2	1.2–1.3	1.2	1.2–1.3
	2011–2015	1.3	1.3–1.4	1.3	1.3–1.3
	2016–2020	1.4	1.3–1.4	1.4	1.4–1.4
Cohort					
	1896–1900	0.5	0.5–0.5	0.5	0.5–0.5
	1901–1905	0.5	0.5–0.5	0.5	0.5–0.5
	1906–1910	0.6	0.6–0.6	0.6	0.6–0.6
	1911–1915	0.6	0.6–0.6	0.6	0.6–0.6
	1916–1920	0.7	0.6–0.7	0.7	0.7–0.7
	1921–1925	0.7	0.7–0.7	0.7	0.7–0.7
	1926–1930	0.7	0.7–0.7	0.7	0.7–0.7
	1931–1935	0.8	0.8–0.8	0.8	0.8–0.8
	1936–1945	0.8	0.8–0.8	0.8	0.8–0.8
	1941–1950	0.9	0.8–0.9	0.9	0.9–0.9
	1946–1951	0.9	0.9–1.0	0.9	0.9–0.9
	1951–1955	1.0	1.0–1.0	1.0	1.0–1.0
	1956–1960	1.1	1.0–1.1	1.1	1.1–1.1
	1961–1965	1.1	1.1–1.2	1.1	1.1–1.1
	1966–1970	1.2	1.1–1.3	1.2	1.2–1.2
	1971–1975	1.3	1.1–1.6	1.3	1.2–1.4
	1976–1980	1.5	0.7–3.0	1.4	1.2–1.7
		Wald Chi-square tests for estimable functions, *p*-value			
Net drift		<0.0001		<0.0001	
All period rate ratios		<0.0001		<0.0001	
All cohort rate ratios		<0.0001		<0.0001	
All local drifts		<0.0001		<0.0001	

DALYs = Disability-Adjusted Life Years; 95% CI = confidence interval. * Results are not shown for burden in aged <40, because no case of death of Alzheimer’s disease and other dementias attributable to high fasting plasma glucose occurred in at least one year in the observed period. Source: Global Burden of Disease study [18].

## Data Availability

Data are contained within the article.
